# Human Umbilical Cord Mesenchymal Stem Cells Inhibit the Function of Allogeneic Activated V*γ*9V*δ*2 T Lymphocytes In Vitro

**DOI:** 10.1155/2015/317801

**Published:** 2015-04-14

**Authors:** Xiaohuan Liu, Ting Feng, Tianxiang Gong, Chongyang Shen, Tingting Zhu, Qihong Wu, Qiang Li, Hong Li

**Affiliations:** ^1^Key Laboratory of Obstetrics & Gynecology and Pediatric Diseases and Birth Defects of Ministry of Education, West China Second Hospital, Sichuan University, Chengdu 610041, China; ^2^Department of Pediatrics, West China Second University Hospital, Sichuan University, Chengdu 610041, China; ^3^Chengdu Blood Center, Chengdu 610041, China

## Abstract

*Background*. Human umbilical cord mesenchymal stem cells (UC-MSCs) can regulate the function of immune cells. However, whether and how UC-MSCs can modulate the function of V*γ*9V*δ*2 T cells has not been fully understood. *Methods*. The PBMCs or V*γ*9V*δ*2 T cells were activated and expanded with pamidronate (PAM) and interleukin-2 (IL-2) with or without the presence UC-MSCs. The effects of UC-MSCs on the proliferation, cytokine expression, and cytotoxicity of V*γ*9V*δ*2 T cells were determined by flow cytometry. The effects of UC-MSCs on Fas-L, TRAIL-expressing V*γ*9V*δ*2 T cells, and V*γ*9V*δ*2 T cell apoptosis were determined by flow cytometry. *Results*. UC-MSCs inhibited V*γ*9V*δ*2 T cell proliferation in a dose-dependent but cell-contact independent manner. Coculture with UC-MSCs reduced the frequency of IFN*γ*+ but increased granzyme B+ V*γ*9V*δ*2 T cells. UC-MSCs inhibited the cytotoxicity of V*γ*9V*δ*2 T cells against influenza virus H1N1 infected A549 cells and also reduced the frequency of Fas-L+, TRAIL+ V*γ*9V*δ*2 T cells but failed to modulate the apoptosis of V*γ*9V*δ*2 T cells. *Conclusions*. These results indicated that UC-MSCs efficiently suppressed the proliferation and cytotoxicity of V*γ*9V*δ*2 T cells and modulated their cytokine production. Fas-L and TRAIL were involved in the regulation. Cell contact and apoptosis of V*γ*9V*δ*2 T cells were not necessary for the inhibition.

## 1. Introduction

Mesenchymal stem cells (MSCs) can spontaneously proliferate and differentiate varieties of cell types, including osteoblasts, chondrocytes, adipoblasts, skeletal myocytes, and tenocyte [1, 2]. Furthermore, previous studies have shown that MSCs have unique immunomodulatory properties and potent immunosuppressive activities because they inhibit alloreactive T cell responses [2–4]. Instead, MSCs have been demonstrated to inhibit the function of immune cells, such as *αβ* T cells [3, 5, 6], NK cells [3, 7–9], macrophage [3], and dendritic cells (DCs) [2, 3, 10]. In addition, MSCs can enhance regulatory T cell responses and help tissue repairs [11]. Because of unique features, they have been widely investigated as a new therapy for graft versus host disease (GvHD) [12, 13], myocardial infarction, stroke, lupus, arthritis, Crohn's disease, acute lung injury, chronic obstructive pulmonary disease (COPD), cirrhosis, multiple sclerosis, amyotrophic lateral sclerosis (ALS), and diabetes [13]. Among all sources of MSCs [2], umbilical cord derived MSCs (UC-MSCs) offer other feasible candidates for MSC-based therapies because of their abundant resources, noninvasive acquiring, low immunogenicity, and great capacity of ex vivo expansion [2, 14].

*γδ* T cells have unique innate and adaptive immunity features and account for approximately 1%–5% of circulating T cells [15, 16]. *γδ* T cells can respond to exotic factors [17, 18] and periphery blood V*γ*9V*δ*2 T cells, and the largest subset of *γδ* T cells can be activated by small nonpeptide phosphoantigens such as isopentenyl pyrophosphate (IPP) and pamidronate (PAM) [19, 20] in an HLA-unrestricted manner [16, 21]. Functionally, V*γ*9V*δ*2 T cells play an important role in host defense, especially in homeostasis and surveillance [21]. Previous studies have shown that V*γ*9V*δ*2 T cells regulate the process of innate immunity [22], immune responses against infection [17, 23], cancer [15, 22, 24], and autoimmune disease [25]. Both V*γ*9V*δ*2 T cells and MSCs play regulating roles in GvHD and some autoimmune diseases [13, 25, 26]; however, how V*γ*9V*δ*2 T cells respond to allogeneic UC-MSCs has not been clarified.

In this study, we isolated and expanded UC-MSCs as well as peripheral blood mononuclear cells (PBMCs) from healthy donors and examined the regulatory effect of allogeneic UC-MSCs on the proliferation, cytokine production, and cytotoxicity of V*γ*9V*δ*2 T cells in vitro. Our findings indicated that UC-MSCs inhibited the proliferation, cytokine production, and cytotoxicity of V*γ*9V*δ*2 T cells in vitro. Cell contact and apoptosis of V*γ*9V*δ*2 T cells were not necessary for the inhibition. Fas-L and TRAIL were involved in the regulation. We discussed the implications of our findings. 

## 2. Materials and Methods

### 2.1. Preparation of UC-MSCs

The experimental protocol was approved by the Institutional Review Board of Sichuan University. UC-MSCs were isolated from human umbilical cords, as described previously [27]. UC-MSCs were cultured in a 75-cm² flask in *α*-MEM medium (Hyclone, Chengdu, China) containing 15% heat inactivated fetal bovine serum (FBS, BioWest, South America), 100 units/mL of penicillin, and 100 *μ*g/mL of streptomycin at 37°C in 5% CO_2_ incubator. When the cells reached 80% confluency, UC-MSCs were digested with 0.25% trypsin and harvested for subculture or irradiation procession. The expanded UC-MSCs were characterized for their abilities to self-renew and differentiate multiple lineages of cells (data not shown). UC-MSCs were irradiated by X-ray (30 Gy) on Precision X-ray, (X-RAD 320), and cocultured with PBMCs or V*γ*9V*δ*2 T cells.

### 2.2. Preparation of PBMCS and V*γ*9V*δ*2 T Cells

Human peripheral blood samples were obtained from healthy donors and PBMCs were isolated by Ficoll-Hypaque (Pharmacia, Piscataway, NJ, USA) density gradient centrifugation, as previously described [23]. The isolated PBMCs were cultured in RPMI-1640 medium supplemented with 10% FBS, 100 units/mL of penicillin, 100 *μ*g/mL of streptomycin, 2 *μ*g/mL of pamidronate disodium (PAM), and 100 IU/mL of human recombinant IL-2 (Invitrogen, Carlsbad, CA, USA). The cells were exposed to freshly prepared PAM and IL-2 medium and then cultured with freshly prepared medium containing IL-2 every three days up to two weeks. The contained V*γ*9V*δ*2 T cells were purified by negative selection using a TCR V*γ*9V*δ*2 T-cell isolation kit, according to the manufacturer's instruction (Miltenyi Biotec, Bergisch Gladbach, Germany). Check the V*γ*9V*δ*2 T cells by flow cytometry. V*γ*9V*δ*2 T cells were gated on CD3+TCR*γδ*+ in lymphocytes.

### 2.3. PBMCs/V*γ*9V*δ*2 T Cells Cocultured with UC-MSCs

PBMCs were cocultured with UC-MSCs in 10% FBS RPMI 1640 medium containing PAM and IL-2 with or without transwell system. PBMCs (1.5∗10^6^/well) were cocultured with 30 Gy-irradiated UC-MSCs at cell ratios of 5 : 1, 20 : 1, 80 : 1, or 320 : 1 for 48 hours to 14 days. In transwell system, PBMCs/V*γ*9V*δ*2 T cells were cultured at the top chamber of 24-well transwell plates (0.4 mm pore; Millipore, Bedford, MA, USA) and 30 Gy-irradiated UC-MSCs were cultured at the bottom chamber of the plates.

### 2.4. Proliferation Assay

The isolated PBMCs (1.0∗10^6^/mL) were labeled with 1 *μ*M 5,6-carboxyfluorescein diacetate N-succinimidyl ester (CFSE, Dojindo, Kumamoto, Japan) for 15 minutes. After being washed, the labeled PBMCs were cocultured with UC-MSCs in triplicate at the cell ratios of 5 : 1, 20 : 1, 80 : 1, and 320 : 1 for 14 days with PAM and IL-2. The cells were exposed to freshly prepared medium every three days. The cells were stained PE-anti-TCR*γδ* and APC-cy7-anti-CD3 (BD Biosciences, San Jose, USA) and the frequency of V*γ*9V*δ*2 T cell proliferation was determined by flow cytometry on a BECKMAN, FC 500, followed by analyzing the data with FlowJo software (Tree Star, Inc., Ashland, OR, USA).

Furthermore, the CFSE-labeled PBMCs (1.5∗10^6^/well) at the top chamber of 24-well transwell plates (0.4 mm pore; Millipore, Bedford, MA, USA) were cocultured with 30 Gy-irradiated UC-MSCs at the bottom chamber of the plates at the cell ratios of 5 : 1, 20 : 1, or 80 : 1 for 14 days with PAM and IL-2. PBMCs alone at the top chamber served as controls. The PBMCs were stained with PE-anti-TCR*γδ* and APC-cy7-anti-CD3 to determine the proliferation of V*γ*9V*δ*2 T cells.

### 2.5. Cytokine Assay

Freshly isolated PBMCs were stimulated with PAM and IL-2 for 12 days and the cells were cocultured with, or without, allogeneic irradiated UC-MSCs at cell ratios of 5 : 1 or 80 : 1 for 48 hours. During the last 4-hour culture, the cells were treated with Brefeldin A. Subsequently, the cells were harvested, stained with APC-cy7-anti-CD3 and APC-anti-TCR*γδ*, fixed, permeabilized, and intracellularly stained with FITC-anti-IFN*γ*, PE-anti-TNF*α*, PE-anti-IL-10, PE-anti-perforin, or FITC-anti-granzyme B (BD Biosciences) to examine the frequency of cytokine-expressing V*γ*9V*δ*2 T cells, respectively.

### 2.6. Cytotoxicity Assay

Human adenocarcinomic alveolar basal epithelial A549 cells provided by the laboratory were cultured in 10% FBS RPMI 1640 medium. Furthermore, A549 cells were cultured overnight and, after being washed with phosphate-buffered saline (PBS), the cells were infected with influenza virus H1N1 (A/PR/8/34) [27] at a multiplicity of infection (MOI) of 2 for 1 hour, followed by washing out free virus with PBS.

Freshly isolated PBMCs were stimulated with PAM and IL-2 for 12 days and were cocultured with allogeneic irradiated UC-MSCs at cell ratios of 5 : 1 or 80 : 1 for 60 hours. The contained V*γ*9V*δ*2 T cells were purified by negative selection, as described above. The purified V*γ*9V*δ*2 T cells (effector) were cocultured in triplicate with influenza virus- (H1N1-) infected A549 cells (target) at an effector-to-target (E/T) ratio of 10 : 1 for 5 hours. Subsequently, the cells were stained with APC-cy7-anti-CD3 and 7AAD (BD Biosciences) and the percentages of dead A549 cells in total CD3-A549 cells were determined by flow cytometry.

### 2.7. Apoptosis Assay

Freshly isolated PBMCs were stimulated with PAM and IL-2 in the presence or absence of irradiated UC-MSCs at the cell ratio of 5 : 1 or 80 : 1 for 14 days. The cells were harvested and stained with APC-cy7-anti-CD3, APC-anti-TCRV*γ*9V*δ*2, and PE-anti-Fas-L or PE-anti-TRAIL. The frequency of Fas-L+ or TRIAL+ V*γ*9V*δ*2 T cells was determined by flow cytometry. In addition, some cells were stained with APC-cy7-anti-CD3, APC-anti-TCR*γδ*, FITC-Annexin-V, and 7-AAD to determine the frequency of apoptotic V*γ*9V*δ*2 T cells.

### 2.8. Statistical Analysis

Data are representative FACS charts or histograms or expressed as the mean ± SEM. The difference among groups was analyzed by ANOVA and post hoc* t*-test using GraphPad Prism software (version 5). A *P* value of <0.05 was considered statistically significant.

## 3. Results

### 3.1. UC-MSCs Inhibit the Proliferation of Allogeneic V*γ*9V*δ*2 T Cells

**Figure 1:**
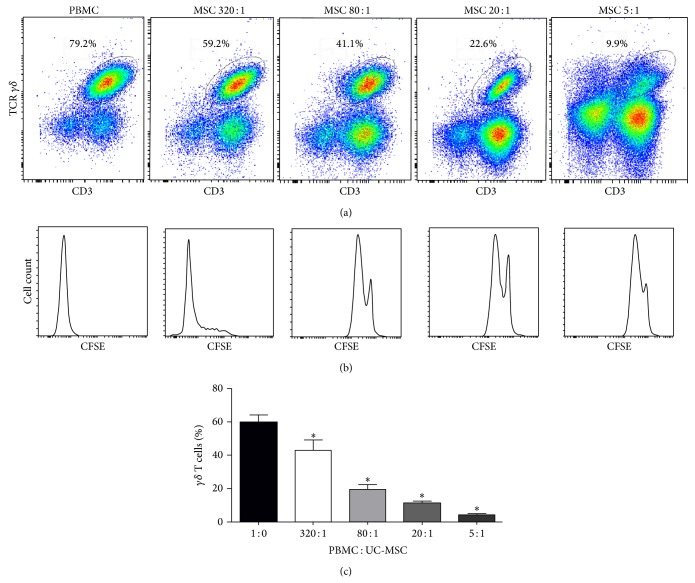
UC-MSCs inhibit *γδ* T cells proliferation in a dose-dependent manner. PBMCs from healthy donors were stained with CFSE (1 *μ*M) and cocultured with, or without, UC-MSCs at the indicated (T : MSCs) ratios for 14 days. The cells were stained with different fluorescent antibodies, as described in in the method section, and the percentages of proliferative *γδ* T cells were determined by flow cytometry. The cells were gated on CD3+TCR*γδ*+ cells and their proliferation was characterized by flow cytometry, followed by quantification. Data are representative charts and histograms or expressed as the means ± SEM of each group of cells from 15 healthy donors. (a) The representative population of CD3+TCR*γδ*+ cells following coculture with, or without, UC-MSCs. (b) The representative histograms of proliferative CD3+TCR*γδ*+ cells. (c) The percentages of proliferative CD3+TCR*γδ*+ cells. ^∗^ *P* < 0.01 versus the controls without coculture with UC-MSCs. UC-MSC: umbilical cord mesenchymal stem cell; PBMC: peripheral blood mononuclear cell; CFSE: 5,6-carboxyfluorescein diacetate succinimidyl ester.

We characterized the frequency of peripheral blood V*γ*9V*δ*2 T cells by flow cytometry analysis and found that peripheral blood V*γ*9V*δ*2 T cells accounted for 3%–8% of T cells (data not shown). Following stimulation with PAM and IL-2 for 14 days, the percentages of V*γ*9V*δ*2 T cells reached 40%–90% of cultured T cells. To determine the effect of UC-MSCs on the allogeneic V*γ*9V*δ*2 T cell proliferation, PBMCs were isolated from healthy donors and labeled with CFSE. The labeled PBMCs were stimulated with PAM and IL-2 in the presence or absence of different numbers of irradiated UC-MSCs for 14 days. Subsequently, the cells were stained with PE-anti-TCR*γδ* and APC-cy7-anti-CD3 and the percentages of V*γ*9V*δ*2 T cells were determined by flow cytometry (Figure 1(a)). Quantitative analysis indicated that while near 60% of V*γ*9V*δ*2 T cells underwent proliferation in the absence of UC-MSCs. The percentages of V*γ*9V*δ*2 T cells in the presence of 320 : 1 UC-MSCs were significantly reduced by 28.3% (*P* < 0.01, Figure 1(c)). The percentages of V*γ*9V*δ*2 T cells in the presence of greater numbers of UC-MSCs were further reduced by 67.2%, 81.7%, and 93.0% for 80 : 1, 20 : 1, and 5 : 1, respectively. Besides, the incomplete dilution of CFSE of V*γ*9V*δ*2 T cells into cell progenies showed the suppression of UC-MSCs on V*γ*9V*δ*2 T cells proliferation (Figure 1(b)). Hence, UC-MSCs inhibited PAM-stimulated V*γ*9V*δ*2 T cell proliferation in a dose-dependent manner in vitro.

**Figure 2:**
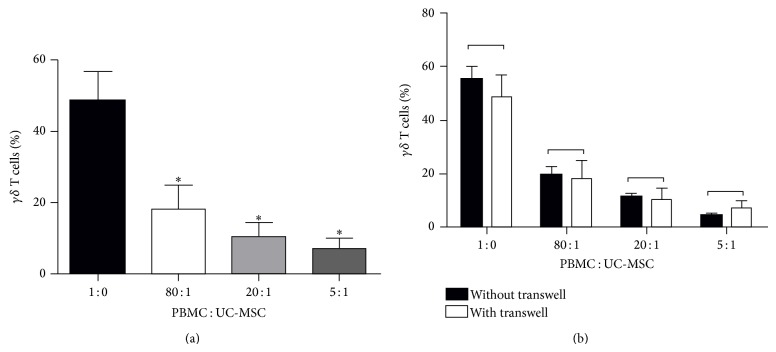
UC-MSCs inhibit the proliferation of *γδ* T cells in a cell-cell contact-independent manner. PBMCs from three healthy donors were labeled with CSFE and cocultured with, or without, UC-MSCs at the different ratios in transwell or together in 6-well plates, followed by simulation with PAM and IL-2 for 14 days. Subsequently, the cells were stained with fluorescent antibodies, as described in the method section. The cells were gated on CD3+TCR*γδ*+ and the percentages of proliferative *γδ* T cells were determined by flow cytometry. Data are expressed as the mean percentages ± SEM of each group of cells from three separate experiments. (a) The percentages of proliferative *γδ* T cells following a separated coculture in transwell plates. (b) The percentages of proliferative *γδ* T cells following coculture in transwell or 24-well plates. ^∗^ *P* < 0.01 versus the controls. UC-MSC: umbilical cord mesenchymal stem cell; PBMC: peripheral blood mononuclear cell.

To understand the importance of cell-to-cell contact in inhibition of UC-MSCs on V*γ*9V*δ*2 T cell proliferation, the CFSE-labeled PBMCs (1.5∗10^6^/well) in the top chamber of 24-well transwell plates were cocultured with irradiated UC-MSCs in the bottom chamber of the plates in triplicate at the cell ratios of 5 : 1, 20 : 1, or 80 : 1 for 14 days. PBMCs alone in the top chamber served as controls. Subsequently, the PBMCs were stained with PE-anti-TCR*γδ* to determine the frequency of V*γ*9V*δ*2 T cells by flow cytometry. As shown in Figure 2(a), UC-MSCs inhibited the frequency of allogeneic V*γ*9V*δ*2 T cells when both types of cells were cocultured together. Similarly, UC-MSCs also inhibited the proliferation of allogeneic V*γ*9V*δ*2 T cells when both types of cells were separately cultured in the top and bottom chambers and there was no significant difference in the inhibitory effects between these two different tests (Figure 2(b)). Together, these data clearly indicated that UC-MSCs inhibited the proliferation of allogeneic V*γ*9V*δ*2 T cells in a cell-to-cell contact independent manner.

### 3.2. UC-MSCs Regulate Cytokine Production by V*γ*9V*δ*2 T Cells

**Figure 3:**
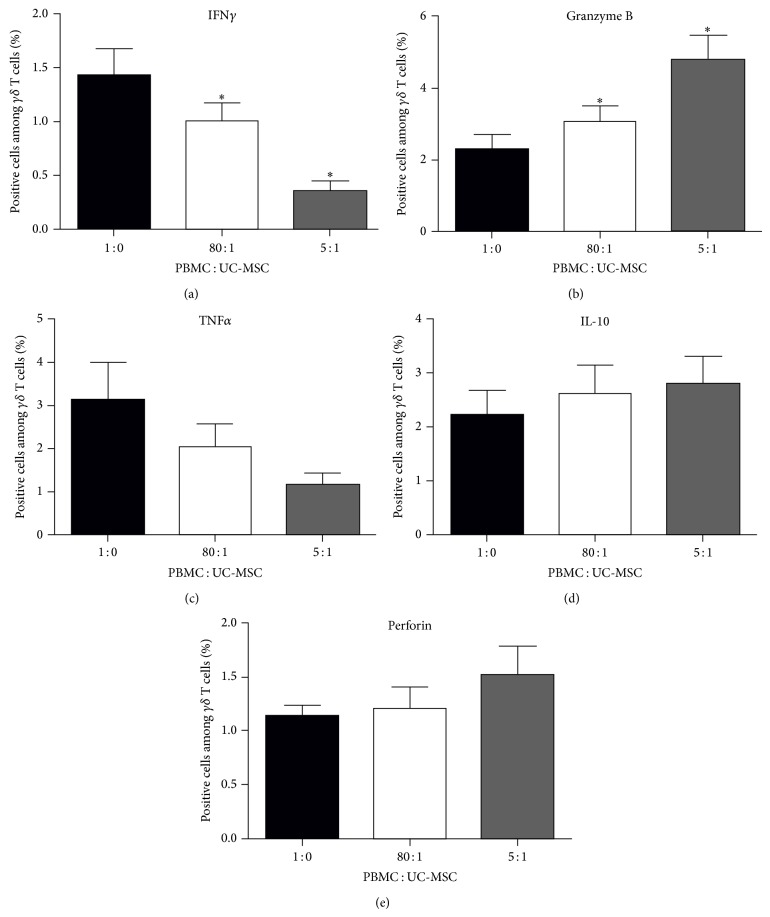
UC-MSCs modulate the expression of cytokines and bioactive effectors in *γδ* T cells. PBMCs were isolated and stimulated with PAM and IL-12 for 12 days. The enriched *γδ* T cells were cocultured with UC-MSCs at the indicated ratios for 48 hours and the cells were stained with different fluorescent antibodies, as described in in the method section. Subsequently, the percentages of IFN*γ*+, granzyme B+, TNF*α*+, perforin+, or IL-10+ *γδ* T cells were characterized by flow cytometry. Data are representative flow cytometry charts or expressed as the mean percentages ± SEM of each group of cells from nine subjects of nine separate experiments. (a–e) Quantitative analysis of the percentages of *γδ* T cells. ^∗^ *P* < 0.05 versus the controls. UC-MSC: umbilical cord mesenchymal stem cell; PBMC: peripheral blood mononuclear cell; TNF*α*: tumor necrosis factor; IFN*γ*: interferon-*γ*; IL-10: interleukin-10.

Activated V*γ*9V*δ*2 T cells can express different cytokines and cytotoxic enzymes. To determine the effect of UC-MSCs on the expression of cytokines and functional enzymes, the isolated PBMCs were stimulated with PAM and IL-2 for 12 days and the cells were cocultured with, or without, irradiated UC-MSCs in triplicate at cell ratios of 5 : 1 or 80 : 1 for 48 hours (with Brefeldin A for the last 4 hours). Subsequently, the cells were harvested, stained with APC-cy7-anti-CD3 and APC-anti-TCR*γδ*, fixed, permeabilized, and intracellularly stained with FITC-anti-IFN*γ*, PE-anti-TNF*α*, PE-anti-IL-10, PE-anti-perforin, or FITC-anti-granzyme B. The percentages of TNF*α*+, IFN*γ*+, perforin+, granzyme B+, or IL-10+ V*γ*9V*δ*2 T cells were determined by flow cytometry. After being gated on CD3+TCR*γδ*+ cells, there was no significant difference in the frequency of TNF*α*+, perforin+, or IL-10+ V*γ*9V*δ*2 T cells in total V*γ*9V*δ*2 T cells regardless of the presence or absence of MSCs (Figures 3(c)–3(e)). In contrast, coculture with UC-MSCs significantly reduced the percentages of IFN*γ*+ V*γ*9V*δ*2 T cells by 29.6% and 75.6% at cell ratios of 80 : 1 and 5 : 1 compared with the control group (*P* < 0.05, Figure 3(a)). However, coculture with UC-MSCs significantly increased the frequency of granzyme B+ V*γ*9V*δ*2 T cells by 34.6% and 109.5% for 80 : 1 and 5 : 1 UC-MSCs compared with control group (*P* < 0.05, Figure 3(b)). The regulatory effects of UC-MSCs trended to be dose-dependent. Hence, UC-MSCs regulated the expression of cytokines and functional enzymes in V*γ*9V*δ*2 T cells.

### 3.3. UC-MSCs Inhibit the Cytotoxicity of V*γ*9V*δ*2 T Cells against Influenza Virus-Infected A549 Cells

**Figure 4:**
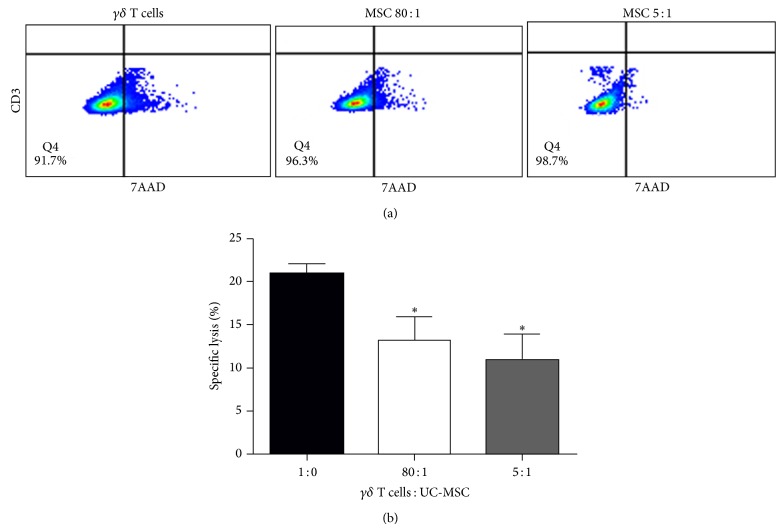
UC-MSCs inhibit the cytotoxicity of activated *γδ* T cells against influenza virus-infected A549 cells in vitro. PBMCs were stimulated with PAM and IL-2 for 12 days and cocultured with, or without, the different ratios of UC-MSCs for 60 hours. Subsequently, the activated *γδ* T cells were purified from the different groups of cells by FACS sorting and the purified *γδ* T cells were cocultured with influenza virus-infected A549 cells at a ratio of 10 : 1 for 5 hours and stained with APC-anti-CD3, FITC-Annexin V, and 7-AAD. The percentages of apoptotic A549 cells were characterized by flow cytometry after gating on CD3-cells. Data are representative flow cytometry charts or expressed as the mean percentages ± SEM of each group of cells from 19 subjects. (a) The representative flow cytometry charts. (b) Quantitative analysis of the percentages of apoptotic A549 cells. ^∗^ *P* < 0.05 versus the controls. UC-MSC: umbilical cord mesenchymal stem cell.

Activated V*γ*9V*δ*2 T cells have cytotoxicity against influenza virus-infected A549 cells [23]. To determine the impact of UC-MSCs on the cytotoxicity of V*γ*9V*δ*2 T cells, the isolated PBMCs were stimulated with PAM and IL-2 for 12 days and were cocultured with, or without, irradiated UC-MSCs in triplicate at cell ratios of 5 : 1 or 80 : 1 for 60 hours. After purification of V*γ*9V*δ*2 T cells, the purified V*γ*9V*δ*2 T cells (effector) were cocultured with influenza virus- (H1N1-) infected A549 cells (target) at an effector-to-target (E/T) ratio of 10 : 1 for 5 hours. Subsequently, the cells were stained with APC-cy7-anti-CD3 and 7AAD, and after being gated on CD3-A549 cells, the percentages of dead A549 cells in total A549 cells were determined by flow cytometry (Figure 4(a)). Coculture of V*γ*9V*δ*2 T cells with UC-MSCs significantly reduced the percentages of dead A549 cells and the inhibitory effects of UC-MSCS on V*γ*9V*δ*2 T cell-mediated cytotoxicity against influenza virus-infected A549 cells trended to be dose-dependent (Figure 4(b)).

### 3.4. UC-MSCs Modulate the Fas-L and TRAIL Expression and Activated V*γ*9V*δ*2 T Cell Apoptosis

**Figure 5:**
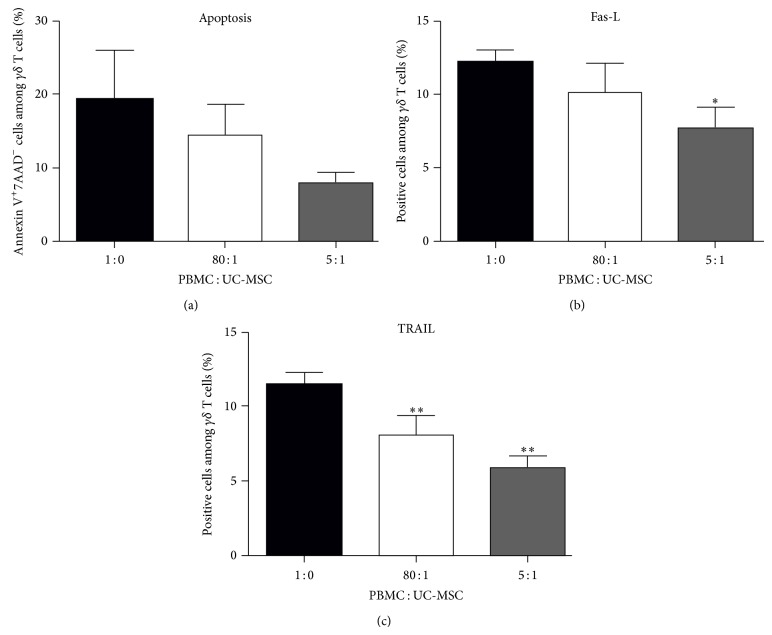
UC-MSCs modulate the expression of Fas-L and TRAIL on *γδ* T cells but do not affect the spontaneous apoptosis of activated *γδ* T cells. PBMCs were cocultured with, or without, the different ratios of UC-MSCs in the presence of PAM and IL-2 for 14 days. The cells were stained with fluorescent antibodies, as described in in the method section. The percentages of apoptotic *γδ* T cells and Fas-L+ or TRAIL+ *γδ* T cells were characterized by flow cytometry analysis. Data are representative flow cytometry analysis of the frequency of apoptotic *γδ* T cells or expressed as the mean percent ± SEM of each group of cells from 19 subjects. (a–c) The quantitative analysis of apoptosis, Fas-L, and TRAIL. ^∗^ *P* < 0.05, ^∗∗^ *P* < 0.05 versus the controls. UC-MSC: umbilical cord mesenchymal stem cell; PBMC: peripheral blood mononuclear cell; Fas-L: Fas ligand; Trail: tumor necrosis factor-related apoptosis-inducing ligand.

Finally, we investigated the impact of UC-MSCs on the apoptosis of activated V*γ*9V*δ*2 T cells. The isolated V*γ*9V*δ*2 T cells were stimulated with PAM and IL-2 in the presence or absence of irradiated UC-MSCs at a ratio of 80 : 1 or 5 : 1 for 14 days. Subsequently, the cells were harvested and stained with APC-cy7-anti-CD3, FITC-Annexin V, and 7-AAD and the percentages of apoptotic cells were determined by flow cytometry. There was no significant difference in the percentages of apoptotic cells among these groups of V*γ*9V*δ*2 T cells (Figure 5(a)). Furthermore, some cells were stained with APC-cy7-anti-CD3, APC-anti-TCRV*γ*9V*δ*2, and PE-anti-Fas-L or PE-anti-TRAIL. The percentages of Fas-L+ or TRAIL+ V*γ*9V*δ*2 T cells were determined by flow cytometry. Quantitative analysis indicated that the percentages of Fas-L+ V*γ*9V*δ*2 T cells when cultured with UC-MSCS at 5 : 1 were significantly lower than that of V*γ*9V*δ*2 T cells without being cocultured with UC-MSCs (Figure 5(b)). Similarly, the percentages of TRAIL+ V*γ*9V*δ*2 T cells cocultured with UC-MSCs were significantly lower than that of V*γ*9V*δ*2 T cells in the absence of UC-MSCs (Figure 5(c)). Therefore, UC-MSCs inhibited Fas-L and TRAIL expression but failed to modulate the spontaneous apoptosis of activated V*γ*9V*δ*2 T cells in vitro.

## 4. Discussion

MSCs have potent immunoregulatory activities and have been tested in the clinical trials for intervention of different inflammatory diseases [13]. UC-MSCs have more advantages than bone marrow-derived ones because of their noninvasive nature and having less immunogenicity as well as powerful proliferative capacity [2, 14]. UC-MSCs have been demonstrated to inhibit the function of *αβ* T cells [3, 5, 6], NK cells [3, 7–9], macrophages [3], and DCs [2, 3, 10] but positively regulate Tregs [11]. In this study, we examined the effect of UC-MSCs on the proliferation and cytotoxicity of V*γ*9V*δ*2 T cells as well as their cytokine and effector expression in vitro. We found that UC-MSCs inhibited the proliferation of PAM/IL-2 stimulated V*γ*9V*δ*2 T cells in a dose-dependent and cell-cell contact-independent manner. Furthermore, we found that UC-MSCs inhibited the expression of IFN*γ* but enhanced granzyme B expression in activated V*γ*9V*δ*2 T cells. In addition, UC-MSCs inhibited the cytotoxicity of activated V*γ*9V*δ*2 T cells against influenza virus-infected A549 cells. The expression of Fas-L and TRAIL on V*γ*9V*δ*2 T cells were inhibited by UC-MSCs as well. However, UC-MSCS failed to modulate the spontaneous apoptosis of activated V*γ*9V*δ*2 T cells. Previous studies have shown that bone marrow-derived MSCs inhibit effector T cell proliferation and IFN*γ* and TNF*α* expression and cytotoxicity against cancer cells [28, 29]. Our data extended previous findings and support the notion that UC-MSCs are powerful inhibitors of T cell immunity. To the best of our knowledge, this was the first report on the regulatory effects of UC-MSCs on the activation and function of human V*γ*9V*δ*2 T cells. Our novel findings may provide new insights into understanding of the regulatory mechanisms underlying the action of UC-MSCs.

In this study, we found that UC-MSCs inhibit the proliferation of PAM/IL-2 stimulated V*γ*9V*δ*2 T cell proliferation in a cell-cell contact-independent manner. While inhibition of T cell proliferation is associated with inducing T cell apoptosis. However, we did not observe that UC-MSCs significantly modulated the apoptosis of activated V*γ*9V*δ*2 T cells in vitro, which may be associated with lower levels of Fas-L and TRAIL expression in V*γ*9V*δ*2 T cells with the presence of UC-MSCs. These findings suggest that UC-MSCs may secrete soluble factors that inhibit V*γ*9V*δ*2T cell proliferation. MSCs can secrete many factors, including transforming growth factor-*β* (TGF-*β*) [30], hepatic growth factors (HGF) [31], prostaglandin E2 (PGE2) [30], IL-10 [30], indolamine 2,3-dioxygenase (IDO) [32, 33], nitric oxide (NO) [30], heme oxygenase-1 (HO-1) [34], and human leukocyte antigen-G (HLA-G) [35], and TGF-*β*, IL-10, and PGE2 are potent inhibitors of T cell immunity [4, 36–38]. These factors together with other unknown factors may act to inhibit T cell proliferation directly and indirectly. We are interested in further investigating what factors positively regulate granzyme B expression in V*γ*9V*δ*2 T cells.

We speculate that the interaction of UC-MSCs with V*γ*9V*δ*2 T cells may be similar to that with NK cells [39]. It is possible that minor allogeneic antigens on UC-MSCS may trigger IFN*γ* V*γ*9V*δ*2 T cell responses and upregulate granzyme B expression, leading to the cytotoxicity against UC-MSCs by the granzyme-NKG2D pathway. Simultaneously, inhibitory factors, such as PGE_2_ [32, 33], TGF-*β* [3], IDO [32, 33], NO [33], and IL-10 [10, 40], secreted by UC-MSCs downregulate the function of V*γ*9V*δ*2 T cells and the serine protease inhibitor 9 (SERPINB9) produced by UC-MSCs attenuates the activity of granzyme B-mediated cytotoxicity. Subsequently, inhibitory factors secreted by UC-MSCs control the function of V*γ*9V*δ*2 T cells by reducing IFN*γ*, Fas-L, and TRAIL expression in V*γ*9V*δ*2 T cells. Immunosuppressive functions of different sources of MSCs are varying and their functions are regulated by many other immunocompetent cells in vivo [41]. Therefore, we are interested in further investigating the precise mechanisms underlying the action of UC-MSCs in regulating the function and survival of V*γ*9V*δ*2 T cells.

Even though the mechanisms underlying the cytotoxicity of V*γ*9V*δ*2 T cells against virus infection are still incompletely understood, the protective role of V*γ*9V*δ*2 T cells has been proved in acute and chronic virus infections. Following the infection with different strains of influenza viruses, V*γ*9V*δ*2 T cells can secrete antiviral cytokines and directly kill virus-infected target cells [42–44], which can be enhanced by phosphoantigen stimulation [44, 45]. UC-MSCs can significantly inhibit the cytotoxicity of V*γ*9V*δ*2 T cells against HIN1 influenza virus in vitro. This result indicates that maybe in viral infection, especially in H1N1 influenza virus infection, UC-MSCs suppress the antiviral protection of V*γ*9V*δ*2 T cells. This needs to be proved in further studies.

In summary, our data indicated that human UC-MSCs inhibited the PAM/IL-2 stimulated V*γ*9V*δ*2 T cell proliferation in a cell-to-cell contact-independent manner and modulated cytokine secretion by V*γ*9V*δ*2 T cells. Furthermore, UC-MSCs inhibited the cytotoxicity of activated V*γ*9V*δ*2 T cells against influenza virus-infected A549 cells. These regulations may be mediated by the inhibition of Fas-L and TRAIL expression byV*γ*9V*δ*2 T cells but not by inducing the apoptosis of V*γ*9V*δ*2 T cells. Our novel data support that UC-MSCs can exhibit immune regulatory capacity by inhibiting V*γ*9V*δ*2 T cells and our findings may provide some clues for further research on the interaction between UC-MSCs and V*γ*9V*δ*2 T cells.
